# 677. To Treat or Not To Treat: Patients with *Clostridioides difficile* PCR-positive/Toxin EIA negative Test Results

**DOI:** 10.1093/ofid/ofad500.739

**Published:** 2023-11-27

**Authors:** Krista Yang, Kaila Cooper, Michael Stevens, Gonzalo Bearman, Alexandra L Bryson, Christopher Doern, L Silvia Munoz-Price, Michelle Doll

**Affiliations:** VCU, Richmond, Virginia; Virginia Commonwealth University Health System, Richmond, VA; West Virginia University, Morgantown, West Virginia; Virginia Commonwealth University Health System, Richmond, VA; Virginia Commonwealth University Health System, Richmond, VA; Virginia Commonwealth University Health System, Richmond, VA; VCUHS, Richmond, Virginia; Virginia Commonwealth University Health System, Richmond, VA

## Abstract

**Background:**

Diagnosis of *C. difficile* infection is complex; multiple diagnostic algorithms are used attempting to identify infected versus colonized patients. Management of symptomatic patients with PCR+ but toxin EIA- disease is controversial, as symptoms are non-specific. Our facility adopted an algorithm using PCR testing first, followed by EIA toxin testing for positive PCRs, in 9/2019. EIA- patients (who are PCR+) are reported as *C. difficile* negative by the lab. We compared outcomes of PCR- patients with those PCR+/EIA-.

**Methods:**

We manually reviewed the patient records of all inpatients with reported negative *C. difficile* results from 9/1/22-11/15/22. Negative results were characterized as either PCR- or PCR+/EIA-. Treatment for a test result was defined as flagyl IV/PO, vancomycin PO, or fidaxomicin given within 7 days of the test (for any reason). We evaluated for repeat *C. difficile* testing, diagnosis of *C. difficile* infection (CDI), death in 60 days, and cause of death. We compared characteristics between patients with EIA+ versus negative status using Chi-square or Fisher’s exact testing in SAS 9.4.

**Results:**

During the study, 321 inpatients had *C. difficile* tests performed that were reported negative. Of these, 267 were PCR-, and 54 were PCR+/EIA-. The proportion of patients treated did not differ between these groups (table 1, p=0.672). Of the 57 patients that had a repeat test within 60 days, 5 were classified positive (PCR+/EIA+): 2 from the PCR+/EIA- group and 3 from the PCR- group (p=0.065). One patient from the PCR- group subsequently had CDI diagnosed at another facility. Five patients in the PCR+/EIA- group and 32 in the PCR- group died within 60 days; *C. difficile* was not implicated in the deaths.

**Figure:**
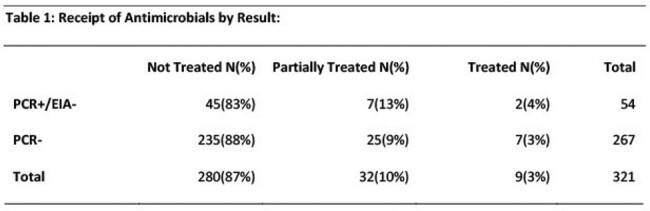

**Conclusion:**

Treatment patterns and clinical outcomes in our facility were similar for patients with *C. difficile* PCR- and PCR+/EIA- diarrhea, in that few patients went on to develop PCR+/EIA+ disease.. Patients were from inpatient locations including bone marrow and solid organ transplant. Study limitations include sample size, and limited 60 day follow up. These data suggest that patients testing PCR+/EIA- may benefit from observation rather than immediate treatment, as 96% (52/54) may not go on to develop CDI. Our findings have implications for lab reporting and treatment of PCR+/EIA- results.

**Disclosures:**

**Christopher Doern, PhD**, GeneCapture: Stocks/Bonds|Quidel: Advisor/Consultant|Shionogi: Honoraria **Michelle Doll, MD, MPH**, Molnlycke: Grant/Research Support

